# Surveillance Strategies After Primary Treatment for Patients with Invasive Lobular Carcinoma of the Breast: Method of Local Recurrence Detection After Breast-Conserving Surgery

**DOI:** 10.1245/s10434-024-15710-1

**Published:** 2024-07-02

**Authors:** Elle N. Clelland, Astrid Quirarte, Harriet T. Rothschild, Mandeep Kaur, Firdows Mujir, Helena Record, Jasmine M. Wong, Rita A. Mukhtar

**Affiliations:** 1 University of California, San Francisco School of Medicine, San Francisco, CA USA; 2 Department of Surgery, University of California, San Francisco, San Francisco, CA USA

**Keywords:** Invasive lobular carcinoma, Recurrence, Breast cancer, Surveillance, Imaging

## Abstract

**Background:**

Invasive lobular carcinoma (ILC) is the second most common subtype of breast cancer. Although mammography is known to have low sensitivity for ILC, there are no data to guide the optimal surveillance after treatment. We explored surveillance strategies after breast-conserving surgery (BCS) for ILC and determined the proportion of imaging-detected recurrences versus interval cancers.

**Methods:**

From an institutional database of 813 women, we retrospectively identified patients who underwent BCS for stage I–III ILC and subsequently had a recurrence. We categorized patients by surveillance strategy and determined the modality of recurrence detection. Interval cancer rates for local recurrences were compared across surveillance strategies using the Chi-square test. We evaluated overall survival with the log-rank test and a Cox proportional hazards model.

**Results:**

We included 58 patients with ILC who had a recurrence after BCS. Of these, 22 (37.9%) had local recurrence, 27 (46.6%) had distant recurrence, and 9 (15.5%) had both local and distant recurrence. Most patients underwent routine mammographic surveillance (65.2%), with 19.6% having supplemental breast magnetic resonance imaging (MRI) and 15.2% having no surveillance. The interval cancer rate was significantly higher in the mammographic surveillance group compared with the MRI surveillance group (61.9% vs. 16.7%; *p* < 0.001).

**Conclusion:**

In this study of patients with recurrence after BCS for primary treatment of stage I–III ILC, we found that most local recurrences were not detected by surveillance mammography. These data support further investigation of supplemental imaging beyond mammography specifically for patients with ILC who undergo BCS.

After completion of treatment for early-stage breast cancer, patients remain at risk for both local and distant recurrence. The magnitude of risk ranges from approximately 3–35% at 10 years depending on tumor biology, stage, and treatment type.^1,2^ For those who experience locoregional recurrence, appropriate treatment can lead to similar overall survival as those without recurrence.^3^ Consequently, surveillance for local recurrence for all breast cancers after breast-conserving surgery (BCS) is recommended, with at least annual mammography and physical examination according to National Comprehensive Cancer Center guidelines.^4–6^

While supplemental imaging with breast magnetic resonance imaging (MRI) is utilized in some cases, its use remains controversial.^5–8^ Although supplemental screening with breast MRI has been shown to have increased cancer detection rates and increased sensitivity over mammography alone in some studies, data also show increased biopsy rates and no demonstrable impact on overall survival.^9–13^ As such, some guidelines cite insufficient evidence to recommend surveillance breast MRI in those with a personal history of breast cancer, while some suggest its use in select groups.^14^ Current guidelines from the American College of Radiology recommend annual supplemental breast MRI for those with a personal history of breast cancer diagnosed under the age of 50 years or in the setting of dense breasts.^11,12,15^

Another potential group of patients who may derive particular benefit from routine breast MRI is those with a prior history of invasive lobular carcinoma (ILC) of the breast.^12^ ILC is the second most common histologic subtype of breast cancer after invasive ductal carcinoma (IDC), comprising 10–15% of all cases.^17^

ILC differs from IDC in several ways, including its clinical presentation, histologic growth pattern, underlying genomic drivers, surgical outcomes, and recurrence pattern.^18^ It is well known that standard imaging techniques have lower sensitivity for detecting ILC, a diffusely growing tumor type.^12,19,20^ Additionally, the characteristic lack of desmoplastic reaction in ILC may make these tumors less likely to form a palpable mass.^21^

We therefore investigated surveillance practices and recurrence detection methods in a cohort of patients treated for early-stage ILC who were subsequently diagnosed with local or distant recurrence. In this analysis, we specifically evaluated the method of detection of local recurrence after BCS in patients initially treated for stage I–III ILC at a single institution. We hypothesized that ILC patients who underwent supplemental surveillance imaging with breast MRI would have a lower interval cancer rate compared with those undergoing mammographic surveillance alone.

## Methods

### Study Population

With Institutional Review Board approval (#22-37379), we conducted a retrospective analysis of a prospectively maintained institutional database containing treatment and outcomes data for consecutive patients with ILC. We identified all patients recorded to have either local or distant recurrence; for this analysis, we included only those who underwent BCS for primary ILC. Local recurrence was defined as biopsy-proven invasive cancer recurrence in the ipsilateral breast or regional lymph nodes.

### Variables

The following data were obtained from the database: age at diagnosis of primary ILC, primary ILC stage (based on American Joint Committee on Cancer 7th Edition), grade, receptor status, type of surgery performed (BCS or mastectomy), and type of recurrence (local, distant, or both). Receptor status was determined by pathology reports from the maintained database (estrogen [ER], progesterone [PR], human epidermal growth factor receptor 2 [HER2]). ER and PR positivity were defined by ≥ 1% staining on immunohistochemistry (IHC). HER2 status was determined by IHC and routine fluorescence in situ hybridization, and type of surgery performed was determined by review of operative reports. We performed an additional chart review to determine what surveillance strategy each patient underwent before the diagnosis of recurrence (no imaging, routine mammography, or supplemental breast MRI). We recorded dates and results of imaging studies and reviewed clinic notes to determine the first modality whereby recurrences were detected. When a patient reported a physical symptom that prompted imaging, a subsequent diagnosis of recurrence was categorized as a ‘palpable’ finding. Similarly, when a finding at clinical breast examination prompted imaging, such cases were also categorized as ‘palpable.’ When recurrences first came to clinical attention due to a finding on a routine scheduled imaging examination, that imaging examination was deemed to be the method of detection and the recurrence was deemed ‘screen detected.’ Recurrences that came to clinical attention via a palpable finding after a normal imaging examination were deemed interval cancers. We calculated the interval cancer rate for the mammographic surveillance group and for the supplemental MRI surveillance group. Additionally, time from prior normal imaging to diagnosis of recurrence was calculated. When available, recurrence histology and local recurrence longest diameter on MRI were recorded.

### Statistical Analysis

**Fig. 1:**
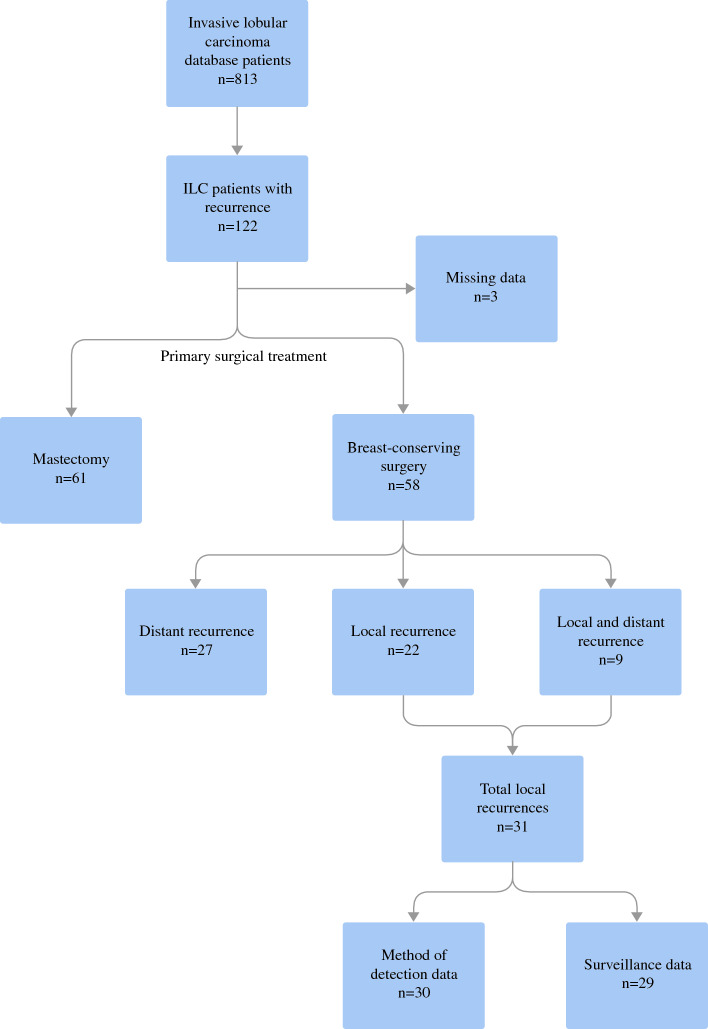
Diagram of the study cohort: patients with local recurrence after breast-conserving surgery for ILC identified from a retrospectively maintained single-institution database. Of note, among those with local recurrence (*n* = 31) the method of recurrence detection was unknown in 1 case, and type of surveillance was unknown in 2 cases. *ILC* invasive lobular carcinoma

We used Chi-square tests for categorical data, and t-tests and analysis of variance for continuous data in Stata 18.0 (StataCorp LLC, College Station, TX, USA) to compare factors associated with each surveillance strategy and to compare the interval cancer rate by surveillance strategy. We used the log-rank test and a Cox proportional hazards model to compare overall survival by surveillance strategy (Fig. 1).

## Results

### Patient Characteristics

Of 813 women with ILC in our institutional database, 122 were diagnosed with breast cancer recurrence. Of those, 61 were treated with mastectomy, 58 had BCS as initial surgery, and 3 were missing surgery data. The 58 patients who underwent BCS for ILC comprise the study cohort. In this group, the average age at initial diagnosis was 60 years (range 28–83 years). At the initial diagnosis of ILC, the mean tumor size was 2.8 cm (standard deviation 2.6 cm) and 42.2% were node-positive (Table 1). Most tumors had ER+/PR+/HER2− receptor subtype (60.0%) and were grade 2 (59.6%). We found that 32.8% of patients received adjuvant chemotherapy and 19.3% received neoadjuvant chemotherapy (Table 1).

**Table 1:** Clinicopathologic characteristics by recurrence type (local recurrence only vs. distant recurrence, which includes those with both distant and local recurrence)

	All cases [*n* = 58]	Local recurrence only [*n* = 22]	Distant ± local recurrence [*n* = 27]	*p* value
Mean age (years)	61.0	57.3	63.7	0.11
ILC size (cm)	2.8	2.1	3.2	0.11
ILC grade				
1	13 (25.0)	5 (25.0)	8 (25.0)	0.24
2	31 (59.6)	14 (70.0)	17 (53.1)	
3	8 (15.4)	1 (5.0)	7 (21.9)	
Tumor stage				
1	29 (51.8)	12 (60.0)	17 (47.2)	0.39
2	16 (28.6)	6 (30.0)	10 (27.8)	
3	11 (19.6)	2 (10.0)	9 (25.0)	
Nodal stage				
0	33 (57.9)	18 (85.7)	15 (41.7)	***0.008***
1	9 (15.8)	1 (4.8)	8 (22.2)	
2	2 (9.5)	2 (9.5)	5 (13.9)	
3	8 (14.0)	0 (0.0)	8 (22.2)	
Receptor subtype				
ER+/PR+/HER−	30 (60.0)	18 (85.7)	12 (41.4)	***0.013***
ER+/PR−/HER−	14 (28.0)	3 (14.3)	11 (37.9)	
ER−/PR−/HER−	4 (8.0)	0 (0.0)	4 (13.8)	
HER2+	2 (4.0)	0 (0.0)	2 (6.9)	
Multifocality	15 (29.4)	6 (35.3)	9 (26.5)	0.51
Lymphovascular invasion	10 (20.8)	2 (12.5)	8 (25.0)	0.32
LCIS present	35 (71.4)	14 (87.5)	21 (63.6)	0.083
Premenopausal	12 (23.5)	7 (36.8)	5 (15.6)	0.084
Adjuvant chemotherapy	19 (32.8)	3 (13.6)	16 (44.4)	***0.015***
Neoadjuvant chemotherapy	11 (19.3)	2 (9.5)	9 (25.0)	0.15

*p* values < 0.05 are shown in bolditalics indicated significant differences between groups

Data are expressed as *n* (%) unless otherwise specified

*ILC* invasive lobular carcinoma, *ER* estrogen receptor, *PR* progesterone receptor, *HER2* human epidermal growth factor receptor 2, *LCIS* lobular carcinoma in situ

### Recurrence Events

**Fig. 2:**
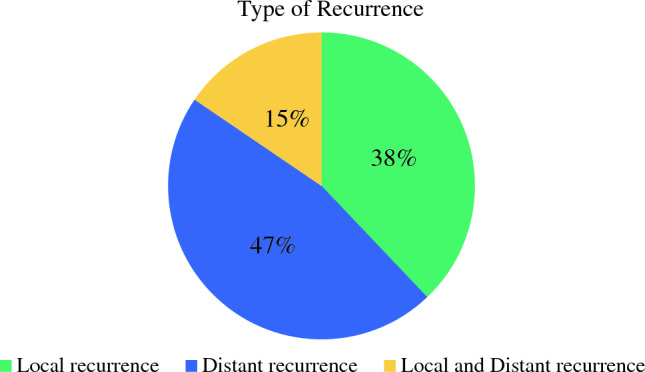
Proportion of each recurrence type (local, distant, or both local and distant)

In this cohort of ILC patients who had recurrence after BCS, 22 (37.9%) had a local recurrence only, 27 (46.6%) had a distant recurrence only, and 9 (15.5%) had both a local and distant recurrence (Fig. 2). In total, there were 31 patients with local recurrence. Primary tumor size was not associated with the type of recurrence, however those with nodal positivity were significantly more likely to have a distant recurrence than local recurrence (58.3% vs. 14.3%; *p* = 0.011). Of those with local recurrence, radiation was utilized in 61.3%, omitted in 29%, and unknown in the remaining cases. Mean time to local recurrence was shorter in those who omitted radiotherapy compared with those who had both lumpectomy and radiation (3.2 years vs. 7.7 years; *p* = 0.05).

### Surveillance Strategies

Of the 58 cases, the method of breast cancer surveillance after primary ILC treatment was known in 46 patients. Most patients (65.2%) underwent routine mammographic surveillance only, while 19.6% underwent supplemental imaging with breast MRI and 15.2% of patients received no surveillance imaging. Those undergoing MRI surveillance were significantly younger at the time of initial ILC diagnosis compared with those undergoing mammography alone, while those receiving no imaging were older at the time of initial ILC diagnosis (mean age 53.8, 62.3, and 72.4 years, respectively; *p* < 0.0091) (Table 2). Although there was no difference in mean primary tumor size across the surveillance modality groups, the seven patients who had no imaging surveillance interestingly had a significantly higher incidence of node positivity compared with those with either mammographic or MRI surveillance (85.7% node-positive vs. 20.7% and 44.4%, respectively; *p* = 0.005) (Table 2).

**Table 2:** Clinicopathologic characteristics of invasive lobular carcinoma cases at initial diagnosis/treatment by surveillance strategy (no imaging, mammographic surveillance, or magnetic resonance imaging surveillance)

	No imaging [*n* = 7]	Mammogram [*n* = 30]	Supplemental MRI [*n* = 9]	*p*-Value
Mean age (years)	74.0	62.5	53.8	***0.0091***
Primary ILC size on surgical pathology (cm)	2.3	2.5	3.3	0.71
Recurrence ILC size on MRI (cm)	1.9	1.6	1.1	0.67
ILC grade				
1	2 (33.3)	4 (13.8)	4 (44.4)	0.37
2	3 (50.0)	21 (72.4)	4 (44.4)	
3	1 (16.67)	4 (13.8)	1 (11.1)	
Tumor stage				
1	3 (42.9)	17 (60.7)	3 (33.3)	0.64
2	3 (42.9)	7 (25.0)	4 (44.4)	
3	1 (14.3)	4 (14.3)	2 (22.2)	
Nodal stage				
0	1 (14.3)	23 (79.3)	5 (55.6)	***0.013***
1	1 (14.3)	4 (13.8)	2 (22.2)	
2	2 (28.6)	1 (3.5)	1 (11.1)	
3	3 (42.9)	1 (3.5)	1 (11.1)	
Receptor subtype				
ER+/PR+/HER−	2 (28.6)	18 (66.7)	6 (66.7)	0.34
ER+/PR−/HER−	4 (57.1)	5 (18.5)	3 (33.3)	
ER−/PR−/HER−	1 (14.3)	2 (7.4)	0 (0.0)	
HER2+	0 (0.0)	2 (7.4)	0 (0.0)	
Multifocality	2 (28.6)	8 (29.6)	1 (14.3)	0.71
Lymphovascular invasion	1 (16.7)	3 (12.0)	3 (37.5)	0.26
LCIS present	3 (50.0)	19 (70.4)	6 (85.7)	0.37
Premenopausal	12 (23.5)	7 (36.8)	5 (15.6)	0.084
Postmenopausal	39 (76.5)	12 (63.7)	27 (84.4)	0.084
Adjuvant chemotherapy	2 (28.6)	10 (33.3)	3 (33.3)	0.97
Neoadjuvant chemotherapy	2 (28.6)	3 (10.0)	3 (37.5)	0.14
Decade of diagnosis (years)				
1980–1990	0 (0.0)	1 (3.3)	0 (0.0)	0.33
1990–2000	0 (0.0)	4 (13.3)	1 (11.1)	
2000–2010	4 (57.1)	7 (23.3)	4 (44.4)	
2010–2020	3 (42.9)	18 (60.0)	3 (33.3)	
2020–2030	0 (0.0)	0 (0.0)	1 (11.1)	

*p* values < 0.05 are shown in bolditalics indicated significant differences between groups

Data expressed as *n* (%) unless otherwise specified

*MRI* magnetic resonance imaging, *ILC* invasive lobular carcinoma, *ER* estrogen receptor, *PR* progesterone receptor, *HER2* human epidermal growth factor receptor 2, *LCIS* lobular carcinoma in situ

### Method of Recurrence Detection and Interval Cancers

Of the 31 patients with a local recurrence, the method of discovery of the local recurrence could be determined for 30 cases. Eight cases (26.7%) were detected by mammogram, 5 (16.7%) were detected by MRI, and 17 (56.7%) cases were detected by palpation. The surveillance strategy prior to diagnosis was known in 29 of these 30 cases, allowing for calculation of the interval cancer rate in those undergoing imaging surveillance.

**Fig. 3:**
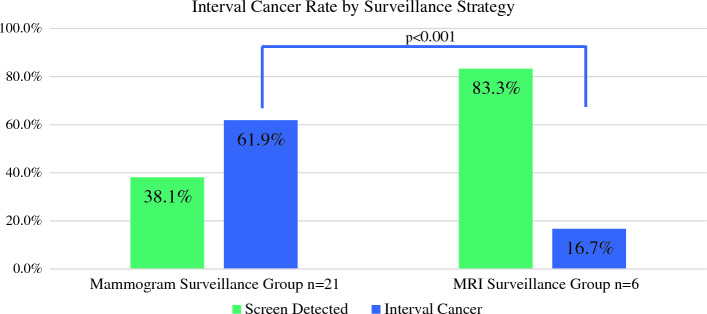
The proportion of local recurrence cases detected by mammography or MRI, or clinically detected (interval cancer) by imaging-surveillance strategy (mammography, MRI). *MRI* magnetic resonance imaging

Among those patients undergoing surveillance imaging, the interval cancer rate was 51.9%, meaning just over half of recurrences were detected clinically (patient symptom or physical examination) instead of on routine imaging. The interval cancer rate was significantly higher in the group having mammography alone compared with those having supplemental breast MRI (61.9% vs. 16.7%; *p* < 0.0001) (Fig. 3). Of the patients having mammographic surveillance, 38.1% (*n* = 8) of local recurrences were detected by mammogram, while the remaining 61.9% (*n* = 13) were interval cancers (detected clinically). Of the patients having MRI surveillance, 83.3% (*n* = 5) of local recurrences were detected by surveillance MRI and the remaining 16.7% (*n* = 1) were interval cancers. Among patients undergoing supplemental breast MRI, no recurrences were detected by mammography (Fig. 3). As expected, in the patients who had no routine imaging surveillance, all local recurrences were detected clinically.

Of the 13 interval cancers that developed in women undergoing mammographic surveillance alone, seven patients had a normal mammogram within 12 months preceding the diagnosis. The remaining six patients had mammographic surveillance more than 12 months before the recurrence diagnosis, ranging from 14.8 to 55.2 months.

In the MRI surveillance group, the only patient with interval cancer had their prior MRI 16.3 months before detection of the palpable tumor.

### Characteristics of Local Recurrence and Overall Survival

The majority (75.9%) of local recurrences were ILC on histology, with the remaining either mixed ILC/IDC or IDC. The size of the local recurrence was only available for 19 patients and was measured radiographically by MRI. There was a non-significant trend toward larger-sized tumors in those detected by palpation, and smaller size in those detected by MRI compared with those detected by mammography (1.9, 1.1, and 1.6 cm, respectively; *p* = 0.67). There was no difference in overall survival among those who underwent mammographic versus supplemental MRI, with a mean follow-up time of 9.2 years (ranging from 0.67 to 29.7 years).

## Discussion

To our knowledge, this is the first study examining surveillance strategies and local recurrence detection methods in patients undergoing BCS for ILC. There are several notable findings from the analysis. First, although mammography is known to have poor sensitivity for patients with ILC, only a minority of patients had supplemental breast MRI as part of their surveillance strategy. The only factor associated with undergoing breast MRI was younger age at primary ILC diagnosis; while breast density was not available in this cohort, the association between younger age and higher breast density raises the possibility that density impacted surveillance imaging recommendations. Indeed, current guidelines from the American College of Radiology suggest adding breast MRI for surveillance in those with a prior history of breast cancer and dense breasts.^16^ Additionally, breast MRI may be utilized for patients who were previously diagnosed with mammographically occult breast cancer. ILC has a higher rate of mammographically occult cancers, as well as a higher rate of understaging on mammography compared with IDC.^10,11^ Because this study lacks an IDC cohort, we cannot compare rates of MRI surveillance by histologic subtype. However, we hypothesize that breast MRI may have been utilized more often in patients with mammographically occult ILC, or in cases with large discrepancies between clinical tumor size and pathologic tumor size.

Interestingly, among those having mammographic surveillance, most local recurrences presented as interval cancers (61.9%). This finding suggests that although ILC is thought to be less likely to form a palpable mass, physical examination (either self-examination or clinical breast examination) did result in the detection of most recurrences in this subset of cases. It is important to note that a lack of adherence to recommended screening guidelines may bias the results against the utility of mammography, as not all patients underwent mammograms yearly. This is reflected in the length of time between the last mammogram and diagnosis of recurrence in several of the interval cancer cases. However, in the group that had supplemental surveillance with MRI, the majority of recurrences were detected by MRI and none were detected by mammography. Together, these findings suggest that routine mammographic screening may have limited utility for surveillance in patients treated for ILC. This finding is somewhat consistent with data showing that in the setting of screening patients without prior breast cancer, mammography has lower sensitivity for detecting ILC.^22^ Because of the poor sensitivity for detection, those with ILC are diagnosed at later stages than those with IDC.

Although this study is limited by a small sample size, the finding that most local recurrences in the breast MRI group were in fact detected by MRI is consistent with prior literature showing a higher cancer detection rate with breast MRI compared with mammogram, although prior studies did not specifically study ILC.^12,21,22^ We were unable to determine biopsy rates and false positive rates because we only included patients who were diagnosed with recurrence. Future analysis of the larger cohort without recurrence may help determine test performance in this study population. Indeed, studies reporting higher rates of biopsies with no impact on overall survival make some clinicians question the benefit of supplemental screening with breast MRI.^22,23^ However, others have argued that because earlier detection of recurrence could lead to better clinical outcomes, the downsides of breast MRI may be tolerable to many patients.^13,24,25^ While it is difficult to argue for surveillance breast MRI for all patients with ILC based on the small number of patients undergoing MRI surveillance in our study, the large difference in the interval cancer rate in the mammography and MRI groups, despite the small cohort, suggests that MRI might have particular utility for those with ILC. Indeed, given the lower sensitivity of mammography specifically in ILC, the potential added benefit of breast MRI is reasonably hypothesized to be greater. Of note, in a recent survey study of radiologists, the majority recommended supplemental screening with breast MRI for those with a personal history of ILC.^22^

The findings in our study suggest that an alternative to conventional mammography is needed for surveillance in ILC patients. One promising solution may be contrast-enhanced mammography, with recent studies showing performance in ILC near that of breast MRI.^26^ This may be a more feasible imaging tool as availability expands. Larger studies are needed both to validate our findings and to investigate novel imaging tools in those with ILC.

The palpable nature of many of the recurrences is reassuring, as ILC is often thought to be non-palpable. This underscores the utility of physical examination and is also reassuring for patients who undergo mastectomy for ILC, where routine imaging for surveillance is not recommended. More data are needed on the rate of palpable tumors in ILC at diagnosis and differences in tumor biology between primary and locally recurrent tumors.

Strengths of this study include the unique nature of these data with a curated review of individual patient charts to determine the surveillance detection method, which can be very difficult to accurately assess since imaging is always obtained to evaluate clinically reported findings. Careful attention was paid to determine the very first presentation of local recurrence, but the retrospective nature of this study raises the possibility that the recurrence detection method was attributed incorrectly in some cases despite careful review. Additionally, because local recurrence occurs in a minority of patients after BCS for ILC, the overall sample size is small. This reduces the statistical power to identify differences in overall survival by surveillance strategy; although we did not find a difference between groups, there is still a possibility that earlier detection of local recurrence could impact both subsequent treatment and long-term outcomes for these patients.

Overall, we found that for patients who undergo BCS after diagnosis of ILC, supplemental imaging surveillance with breast MRI is more common in younger women and is associated with significantly lower interval cancer rate compared with mammography alone. These findings suggest that surveillance MRI may have utility for those with a prior personal history of ILC, which should be validated in larger studies.

## Conclusion

Guidelines for screening surveillance after BCS are not histology specific and may not be optimized for the histological growth pattern and presentation of recurrences in ILC. Our study suggests that supplemental imaging for surveillance should be considered specifically for those after BCS for ILC, and questions the utility of routine mammography.
